# DC-SIGN receptor is expressed by cells from cutaneous leishmaniasis lesions and differentially binds to *Leishmania* (*Viannia*) *braziliensis* and *L.* (*Leishmania*) *amazonensis* promastigotes

**DOI:** 10.1590/0074-02760220044

**Published:** 2023-03-27

**Authors:** Carolina de O Mendes-Aguiar, Milene Yoko Kitahara-Oliveira, Ana Cristina Oliveira de Almeida, Marcia Pereira-Oliveira, Manoel Paes de Oliveira Neto, Claude Pirmez, Elizabeth Pereira Sampaio, Adriano Gomes-Silva, Alda Maria Da-Cruz

**Affiliations:** 1 Fundação Oswaldo Cruz-Fiocruz, Instituto Oswaldo Cruz, Laboratório Interdisciplinar de Pesquisas Médicas, Rio de Janeiro, RJ, Brasil; 2 Universidade Federal do Rio Grande do Norte, Instituto de Medicina Tropical do Rio Grande do Norte, Natal, RN, Brasil; 3 Universidade do Estado do Rio de Janeiro, Faculdade de Ciências Médicas, Departamento de Microbiologia, Imunologia e Parasitologia, Rio de Janeiro, RJ, Brasil; 4 Fundação Oswaldo Cruz-Fiocruz, Instituto Nacional de Infectologia Evandro Chagas, Laboratório de Pesquisa Clínica em Micobacterioses, Rio de Janeiro, RJ, Brasil; 5 National Institute of Allergy and Infectious Diseases, Laboratory of Clinical Immunology and Microbiology, Bethesda, MD, USA; 6 Fundação Oswaldo Cruz-Fiocruz, Instituto Oswaldo Cruz, Laboratório de Hanseníase, Rio de Janeiro, RJ, Brasil

**Keywords:** DC-SIGN, cutaneous leishmaniasis, inflammatory infiltrate, Leishmania (Viannia) braziliensis, Leishmania (Leishmania) amazonensis

## Abstract

**BACKGROUND:**

Dendritic cells (DCs) specific intercellular adhesion molecule (ICAM)-3-grabbing non integrin receptor (DC-SIGN) binds to subgenera *Leishmania* promastigotes mediating its interaction with DC and neutrophils, potentially influencing the infection outcome.

**OBJECTIVES:**

In this work, we investigated whether DC-SIGN receptor is expressed in cells from cutaneous leishmaniasis (CL) lesions as well as the *in vitro* binding pattern of *Leishmania* (*Viannia*) *braziliensis* (Lb) and *L.* (*L.*) *amazonensis* (La) promastigotes.

**METHODS:**

DC-SIGN receptor was labeled by immunohistochemistry in cryopreserved CL tissue fragments. *In vitro* binding assay with CFSE-labeled Lb or La promastigotes and RAJI-transfecting cells expressing DC-SIGN (DC-SIGN^POS^) or mock-transfected (DC-SIGN^NEG^) were monitored by flow cytometry at 2 h, 24 h and 48 h in co-culture.

**RESULTS:**

In CL lesion infiltrate, DC-SIGN^POS^ cells were present in the dermis and near the epidermis. Both Lb and La bind to DC-SIGN^POS^ cells, while binding to DC-SIGN^NEG^ was low. La showed precocious and higher affinity to DC-SIGN^hi^ population than to DC-SIGN^low^, while Lb binding was similar in these populations.

**CONCLUSION:**

Our results demonstrate that DC-SIGN receptor is present in *L. braziliensis* CL lesions and interact with Lb promastigotes. Moreover, the differences in the binding pattern to Lb and La suggest DC-SIGN can influence in a difference way the intake of the parasites at the first hours after *Leishmania* infection. These results raise the hypothesis that DC-SIGN receptor could participate in the immunopathogenesis of American tegumentary leishmaniasis accounting for the differences in the outcome of the *Leishmania* spp. infection.

Cutaneous leishmaniasis (CL) due to *Leishmania* (*Viannia*) *braziliensis* is the most common clinical form of leishmaniasis in Brazil.^1^ Skin CL lesions are characterized by a chronic granulomatous inflammatory infiltrate, with lymphocytes, plasmocytes, and macrophages.^2^ The presence of dendritic cells (DC) in CL lesions has been known since the 1980’s.^3^ DCs are located in the epidermis, being capable to migrate to the dermis, and to uptake *Leishmania* amastigotes through the Fcγ receptor.^4^ ^,^ ^5^ In CL lesions, DCs can be found harboring *Leishmania* amastigotes.^6^ ^,^ ^7^ However, infected DCs transport the parasite to draining lymph nodes and initiate the adaptative immune response.^4^ In the course of the infection, DCs producing IL-12 can be detected at the beginning of the inflammatory process.^8^ Moreover, DCs, professional antigen presenting cells, prime *Leishmania* specific CD4^+^ and CD8^+^ T cells and maintain the T cell memory activation.^9^ However, depending on *Leishmania* species, different DC subpopulations are mobilized, which in turn influences the course of infection.^10^ ^,^ ^11^ It was shown that receptor signaling also contributes to the differentiation of protective inflammatory DC in *L. (V.) braziliensis* infection.^12^

DC specific intercellular adhesion molecule (ICAM)-3-grabbing non-integrin receptor (DC-SIGN) is a member of the C-type lectin receptors family, also known as CD209.^13^ DC-SIGN is expressed by immature DCs and macrophages in lymphoide organs and peripheral tissues.^14^ In normal skin, dermal DC, with the appearance of macrophages cells, express CD209 receptor.^15^ Many functions are associated with DC-SIGN receptor. The presence of DC-SIGN in DCs facilitates rolling and trans-endothelial migration by binding to ICAM-2 ligand.^16^ DC-SIGN receptor in DCs surface interacts with ICAM-3 present in T cell membrane, suggesting that these molecules participate and stabilize the contact between DCs and T cells.^13^ DC-SIGN receptor can act as a pathogen-recognized receptor, which recognized specific carbohydrates present in the surfaces of pathogens. Many pathogens utilize DC-SIGN receptor to invade DCs and escape the immune system.^17^ In HIV infection, DC-SIGN acts as a receptor for HIV, capturing the virus and transmitting it to a targeted T cell.^13^ *Mycobacterium tuberculosis* binds with high affinity to DC-SIGN receptor and can be uptaken DCs via this receptor. *In vivo*, *M. tuberculosis* antigens were detected in DC-SIGN positive cells.^18^ In lepromatous leprosy lesions, DC-SIGN positive cells harbor *M. leprae* bacillus.^19^ In non-infectious cutaneous inflammatory disease, like psoriasis, DC-SIGN positive cells can be detected in skin lesions. The expression levels of DC-SIGN receptor in inflammatory infiltrate were significantly higher in lesions than in normal skin, suggesting an involvement of this receptor in psoriasis pathogenesis.^20^ ^,^ ^21^ Both in tegumentary or visceral leishmaniasis, the related species can bind to DC-SIGN receptor.^22^ ^,^ ^23^ Recently, it was shown that interaction between polymorphonuclear cells and *L.* (*L.*) *amazonensis* intermediated by DC-SIGN receptor is required for the release of inflammatory mediators.^24^ In this report, we investigated whether DC-SIGN receptor is expressed in CL lesions caused by *L.* (*V.*) *braziliensis*, and explored the involvement of DC-SIGN on the initial infection process of two species with epidemiological importance in the Americas, *L.* (*V.*) *braziliensis* (Lb) and *L.* (*L.*) *amazonensis* (La) promastigotes.

## SUBJECTS AND METHODS

*Patients* - We evaluated six CL patients who had acquired the disease in Rio de Janeiro, Brazil, which is an endemic area for *L. (V.) braziliensis* infection. All of them were men, mean age 39.2 ± 10.6 years old. The mean period of illness was 35.7 ± 19.8 days and the lesions were ulcerated. The Montenegro skin test was positive in five out six cases (mean 18.4 ± 5.8 mm). The polymerase chain reaction (PCR) assay to detect *Leishmania* kDNA was positive in all of them. Patients were successfully treated with antimonial pentavalent as recommended by the Brazilian Ministry of Health. Informed consent was obtained from each subject, and a skin biopsy was performed for diagnostic purposes. The skin fragment was cryopreserved in optimal cutting temperature (OCT) resin blocks at -196ºC (Tissue Tek; Sakura Finetek, Torrance, CA, USA) until the time of use. All procedures were approved by the Ethical Committee of the Fundação Oswaldo Cruz (CEP FIOCRUZ no. 291/05; CEP IPEC no.390/07), Ministério da Saúde, Rio de Janeiro, Brazil.

*RAJI cells and Leishmania culture* - RAJI-transfected cells expressing DC-SIGN (DC-SIGN^POS^) and RAJI-mock transfected (DC-SIGN^NEG^) cells (Sigma Chemical Company, Saint Louis, USA) were maintained in culture in RPMI-1640 medium supplemented, with 2 mM L-glutamine (Gibco BRL, Gaithersburg, Germany), 100 U/mL penicillin (Sigma, USA), 100 µg/mL streptomycin (Sigma, USA) and 10% fetal bovine serum (FBS; Gibco BRL, Germany) (complete medium), at 37ºC in a humidified CO_2_ incubator.

Promastigotes of *L.* (*V.*) *braziliensis* (MCAN/BR/1998/619) and *L.* (*L.*) *amazonensis* (MHOM/BR/75/Josefa) were grown at 26ºC in Scheneider’s insect medium (Sigma, USA), supplemented with 2 mM L-glutamine (Gibco BRL, Germany), antibiotics (100 U/mL penicillin and 100 µg/mL streptomycin (Sigma, USA) and 10% FBS (Gibco BRL, DE). Parasites were grown for four days in culture and used to perform the binding assay at stationary phase.

*Leishmania - RAJI cell binding assay* - RAJI (DC-SIGN^NEG^ and DC-SIGN^POS^) cells and carboxyfluorescein succinimidyl ester (CFSE) (Celltrace^TM^ CFSE cell proliferation kit; Life Technologies, USA)-labeled promastigotes were adjusted to 1 cell:5 promastigotes in 24 well-plates containing RPMI-1640 complete medium and incubated at 37ºC in a humidified CO_2_ incubator. The binding assay was monitored for 2, 24 and 48 h. Later on, the co-cultured cells were collected and processed for flow cytometry.

*Flow cytometry analysis for binding assay* - RAJI cells (DC-SIGN^NEG^ and DC-SIGN^POS^) bound to *Leishmania* were collected from culture and washed with cold phosphate-buffered saline (PBS) containing 0·01% sodium azide (NaN3; Sigma, USA) and 5% FBS (PBS-Az/FBS). After that, 3 x 10^6^/mL RAJI (DC-SIGN^NEG^ and DC-SIGN^POS^) cells were incubated for 30 min at 4ºC in presence of 10 µL of mouse anti-human DC-SIGN monoclonal antibody (R&D Systems, Minneapolis, USA). After incubation, the cells were washed in PBS-Az/FBS and incubated with goat anti-mouse PC7 secondary antibody (Santa Cruz Biotechnology, Texas, USA) for 30 min at 4ºC. The cells were fixed in a fixing solution containing 1% paraformaldehyde in PBS (PBS-PF 1%) for 20 min at 4ºC. The cells were then washed with PBS-PF 1% and resuspended in PBS-Az prior to analysis. The *Leishmania* promastigotes were labelled with carboxyfluorescein succinimidyl ester (CFSE, Invitrogen), which does not interfere in the parasite viability and infectivity, to be detected by flow cytometry assay. For flow cytometry analysis, 30.000 events in total lymphocyte gate (R1) per sample were acquired in a fluorescence activated cell sorter (CyAn ADP analyzes, Beckman Coulter). DC-SIGN surface receptor and CFSE-labelled *Leishmania* promastigote were analyzed using Summit 4.3 software. The total lymphocyte gate (R1) was settled based on size (forward scatter: FSC) and granularity (side-scatter: SSC). RAJI (DC-SIGN^NEG^ and DC-SIGN^POS^) cells were observed in lymphocyte gate. For binding assay analysis, a dot-plot graphic (DC-SIGN/PE-Cy7 x CFSE) gated in R1 was created, and the percentage results are observed in double positive quadrant. The results were expressed as a percentage mean of positive cells. Five independent experiments were performed.

*Immunohistochemistry* - To detect surface DC-SIGN receptors, the slides containing cells from CL lesions were fixed in acetone PA (Merck, Darmstadt, Germany/DE) and hydrated in phosphate-buffered saline (PBS) pH 7.4. The specimens were incubated with anti-human DC-SIGN (1:40; R&D Systems, USA). Dako Envision system (DakoCytomation, Carpinteria, CA, USA) was used to link anti-mouse primary antibodies to substrate. The staining was completed using 3-amino-9-ethylcarbazole (AEC; Sigma, USA) as the substrate-chromogen system. The slides were counterstained with Mayer’s hematoxylin (Merck, DE). For control, in the first step, antibody was omitted. The slides were examined under a light microscope (Nikon, Eclipse E600, Japan) with Cool Snap-Pro Color camera and acquired by ImagePro^®^ Program (Media Cybernetics, Inc., USA). Only cells with visible nucleus and red-brown immune stain were counted as positive cells. All fields were counted in each section at a magnification of 1000x. The size of the section was measured using a millimeter paper. 

*Statistical analysis* - Statistical analysis was performed by One-way or Two-way analysis of variance (Anova) tests using the GraphPad Prism software version 5·00 for Windows (GraphPad Software, San Diego, CA, USA). The results were expressed as the mean ± standard error or mean ± standard deviation. A p value ≤ 0.05 was considered significant.

## RESULTS

**Fig. 1:**
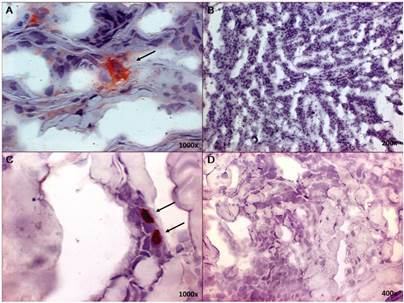
DC-SIGN positive cells in inflammatory infiltrate (arrows) of cutaneous leishmaniasis lesions caused by *Leishmania* (*Viannia*) *braziliensis*. Slide were stained with anti-human CD209 (A and C) followed by Dako Envision System. The slides were then developed with 3,3-diaminobenzidine *(red-brown)* and counterstained with Mayer’s hematoxylin. For control propose, anti-human CD209 antibody was omitted (B and D). Original magnification: 1000x (A and C), 400x (D) and 200x (B).

*Cells expressing DC-SIGN receptor were present in cutaneous leishmaniasis lesions* - We evaluated the expression of DC-SIGN in the inflammatory cell infiltrate of American CL lesions due to *L.* (*V.*) *braziliensis*. The patients evaluated herein presented a clinical prolife similar to that observed in Rio de Janeiro State.^25^ We observed DC-SIGN^POS^ cells (stained in red-brown color) were present in CL lesions (Fig. 1, panel A and C). The DC-SIGN^POS^ cells were located in the dermis and near the epidermis, isolated or in groups, and had similar morphology to macrophages. This indicates that this receptor can be used to uptake parasite forms. In the next step, we evaluated whether Lb and La promastigotes interact with DC-SIGN.

**Fig. 2:**
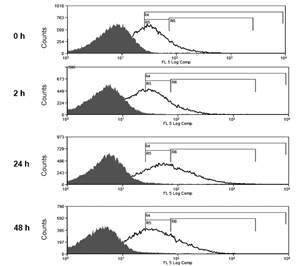
representative flow cytometry histogram of DC-SIGN receptor analysis in RAJI cells in different time points of cell culture. Electronic gates R4 represents total RAJI transfected with DC-SIGN cells expressing DC-SIGN, R5 represents RAJI DC-SIGN^POS^ cells expressing low amount of DC-SIGN (DC-SIGN^Low^), and R6 represents RAJI DC-SIGN^POS^ cells expressing high amount of DC-SIGN (DC-SIGN^Hi^).

*DC-SIGN expression in RAJI cells membranes* - To determine the best day to perform the binding assay, we monitored the RAJI cells proliferation and DC-SIGN receptor expression during three days of culture (Fig. 2). During these three days in culture, the mean percentage of the DC-SIGN receptor expression in the surface of the RAJI^POS^ was not significantly different (0h: 61.5% ± 2.1%; 2h: 64.9% ± 6.0%; 24 h: 73.0% ± 2.7%; 48 h: 65.8% ± 1.8%; ANOVA p = 0.23) (Table).

**TABLE:** DC-SIGN expression in RAJI cells transfected or not with DC-SIGN during the 48 h in culture

	DC-SIGN expression in RAJI cells		
Time points of RAJI cell cultures	DC-SIGN^Neg^ (%)	DC-SIGN^Pos^ (%)	DC-SIGN^Hi^ (%)
0 h	3.1 ± 0.7	61.5 ± 2.1	18.8 ± 3.6
2 h	3.3 ± 0.7	64.9 ± 6.0	26.2 ± 4.5
24 h	3.2 ± 0.5	73.0 ± 2.7	43.2 ± 5.7*
48 h	4.2 ± 1.0	65.8 ± 1.8	26.2 ± 5.5

Five independent experiments were performed. Results are expressed by means ± SEM. DC-SIGN - dendritic cells specific ICAM-3-grabbing non integrin receptor; Neg: negative; Pos: positive; Hi: high positive. *p < 0.05

The flow cytometry analysis showed that RAJI DC-SIGN^POS^ cell population can be divided into two minor populations, based on the intensity of DC-SIGN receptor expression, DC-SIGN^Low^ or DC-SIGN^Hi^ (Fig. 2). Electronics gates were constructed to select RAJI^POS^ (R4), DC-SIGN^Low^(R5) or DC-SIGN^Hi^ (R6) cell populations (Fig. 2). After 24 h in cell culture, DC-SIGN^POS^ cell population was significantly enriched of DC-SIGN^Hi^ cell population (43.2% ± 5.7%; p = 0.05), when compared with other time points analyzed (0 h: 18.8% ± 3.6%; 2 h: 26.2% ± 4.5%; 48 h: 26.2% ± 5.5%) (Fig. 2 and Table).

*American Leishmania species bind in RAJI DC-SIGN positive cells* - To determine whether DC-SIGN receptor binds to *Leishmania* dermothropic species, we evaluated two species from subgenera *Viannia* and *Leishmania* that have epidemiological importance in America*.* For that, CFSE labelled *L.* (*V.*) *braziliensis* or *L.* (*L.*) *amazonensis* promastigotes were co-cultured with RAJI DC-SIGN positive and negative cells. The binding receptor percentage was evaluated by flow cytometry over different times from 0 h to 48 h. RAJI cells did not internalize *Leishmania* promastigotes.

**Fig. 3:**
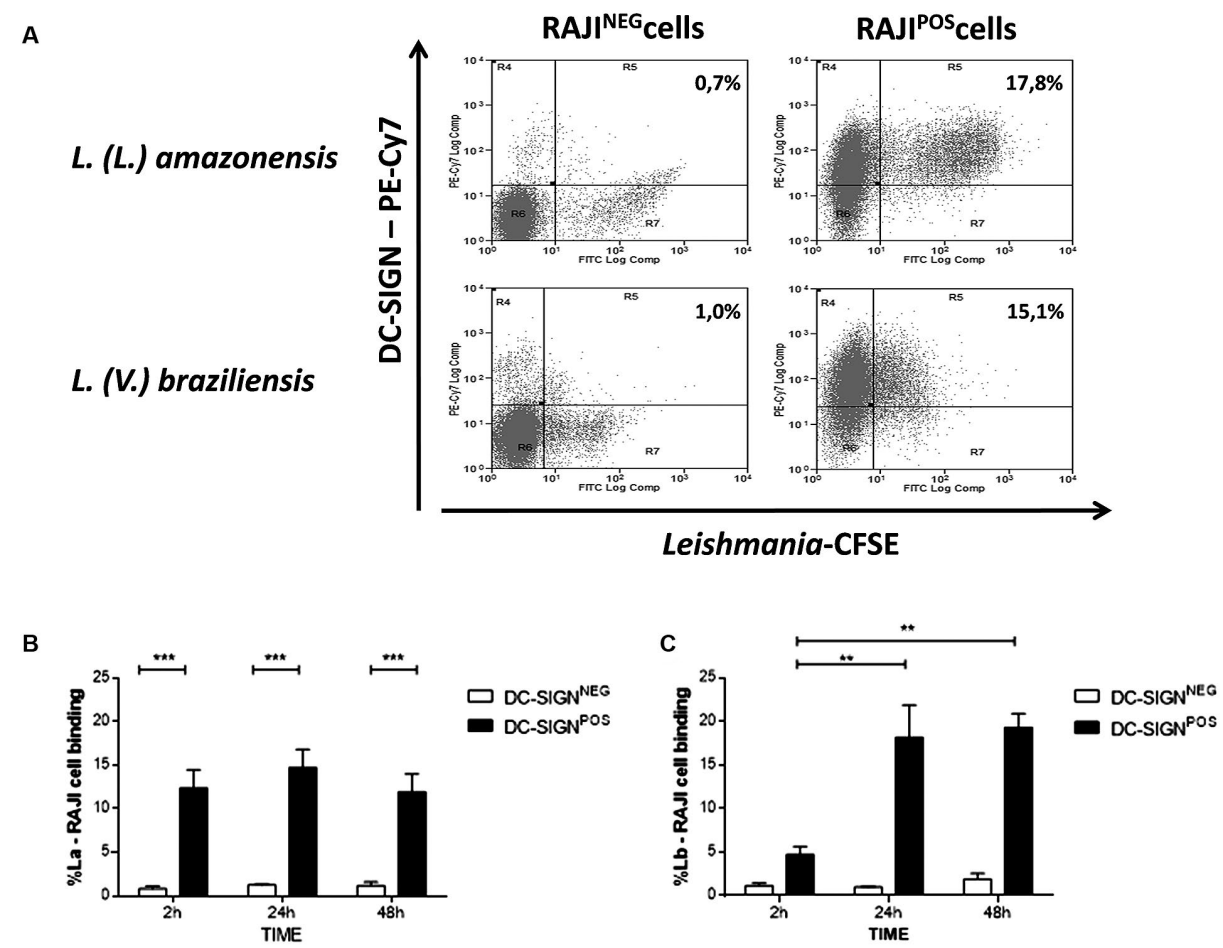
binding analysis of *Leishmania* (*L.*) *amazonensis* (La) or *L.* (*Viannia*) *braziliensis* (Lb) promastigotes to RAJI DC-SIGN^NEG^ or RAJI DC-SIGN^POS^ cells after 2-48 h of co-culture. (A) Dotplot to double-staining analysis of *Leishmania*-CFSE (x axis) and DC-SIGN (CD209)-PE-Cy7 (y axis) from DC-SIGN^NEG^ (left panels) or DC-SIGN^POS^ (right panels) cells after 24 h of co-culture. The percentage of *Leishmania*-RAJI cells binding in different time points were analised for *L.* (*L.*) *amazonensis* (B) or *L.* (*V.*) *braziliensis* (C). * p < 0.05; ***p < 0.001.

Both Lb and La promastigotes presented high binding interaction with the DC-SIGN^POS^ cells, while the interaction with the DC-SIGN^NEG^ cell was low (Fig. 3). After 2 hours in co-culture, the percentage of interaction between La and the DC-SIGN^POS^ cells was significantly higher (12.4% ± 2.11%; p < 0.001) when compared to DC-SIGN^NEG^ (0.81% ± 0.30%) (Fig. 3A-B). The binding between La and DC-SIGN^POS^ cells was maintained even at 48 h of interaction (24 h - DC-SIGN^POS^: 14.7% ± 2.07%; DC-SIGN^NEG^: 1.23% ± 0.15% p < 0.001; 48 h - DC-SIGN^POS^: 11.9% ± 2.03%; DC-SIGN^NEG^: 1.17% ± 0.41%; p < 0.001) (Fig. 3B). For Lb, no binding differences was observed at 2 h of co-culture in the presence or absence of the DC-SIGN receptor (DC-SIGN^POS^: 4.7% ± 0.94%; DC-SIGN^NEG^: 1.17% ± 0.27%; p > 0.05). The interaction between Lb and DC-SIGN receptor was observed only at 24 h in co-culture, maintaining the same binding levels at 48 h (24 h - DC-SIGN^POS^: 18.2% ± 3.7%; DC-SIGN^NEG^: 1% ± 0.05%; p < 0.001; 48 h - DC-SIGN^POS^: 19.3% ± 1.58%; DC-SIGN^NEG^: 1.9% ± 0.66%; p < 0.001) (Fig. 3C).

**Fig. 4:**
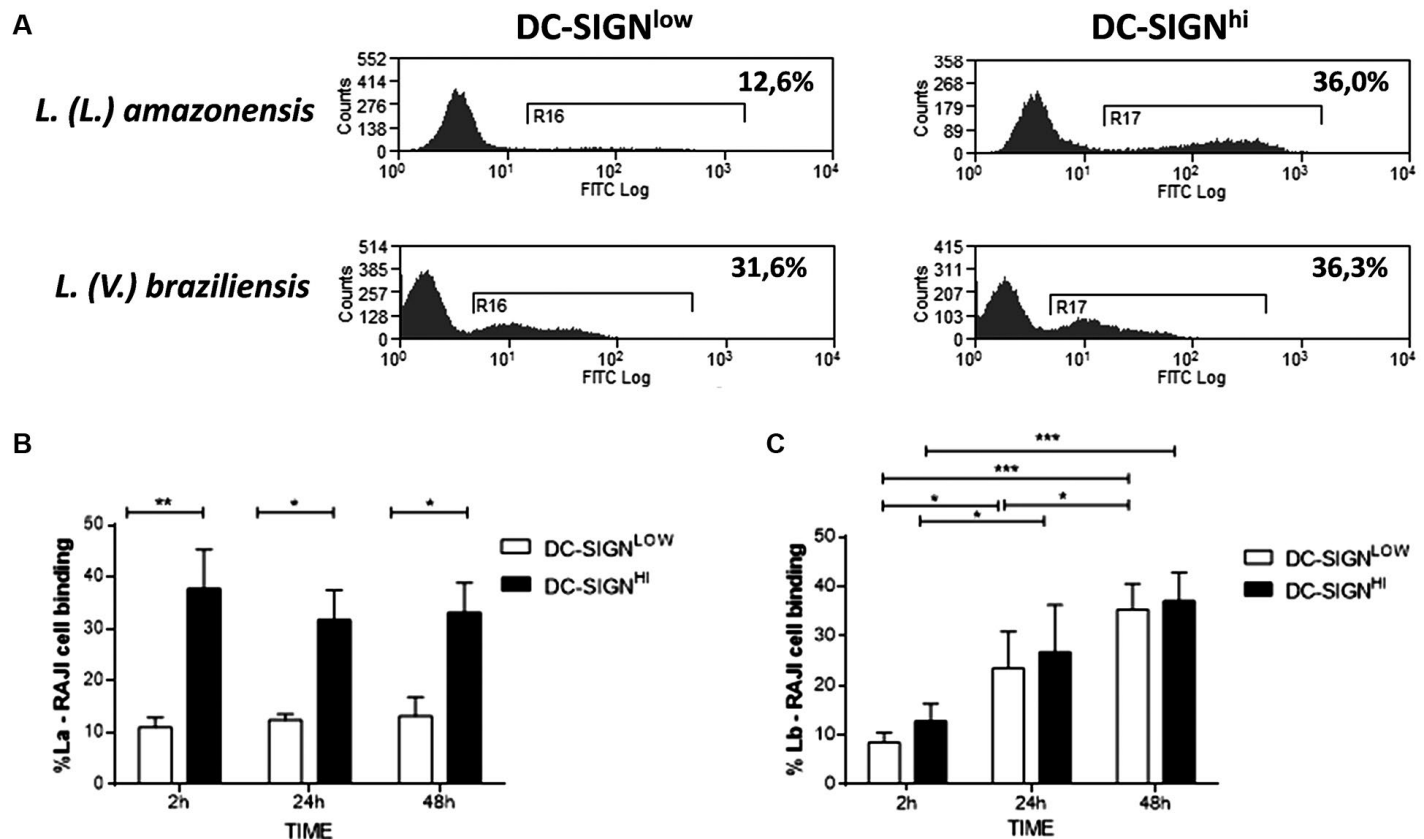
binding analysis of *Leishmania* (*Leishmania*) *amazonensis* (La) or *L.* (*Viannia*) *braziliensis* (Lb) in RAJI DC-SIGN^Low^ or RAJI DC-SIGN^Hi^ populations after 2-48 h of co-culture. (A) Representatives histograms analysis of *Leishmania*-CFSE in DC-SIGN^Low^ (left panels) or DC-SIGN^Hi^ (right panels) after 24 h of co-culture. The percentage of *Leishmania*-CFSE binding to DC-SIGN^Low^ or DC-SIGN^Hi^ gated populations in different time points were analyzed for *L.* (*L.*) *amazonensis* (B) or *L.* (*V.*) *braziliensis* (C). ^*^ p < 0.05; ^**^ p < 0.01; ^***^ p < 0.001.

*Leishmania (L.) amazonensis promastigotes bind preferentially to RAJI DC-SIGN* ^*Hi*^ , *while L. braziliensis promastigotes bind similarly to DC-SIGN* ^*Hi*^ *or* ^*Low*^ *RAJI cells* - To verify whether the *Leishmania* parasites bind specifically to cells with DC-SIGN receptor, we analyzed the binding of *Leishmania* to two RAJI DC-SIGN^POS^ populations: DC-SIGN^Hi^ or DC-SIGN^Low^ (Fig. 4A). The percentage of binding between La and the DC-SIGN^Hi^ population was significantly higher and constant (2 h: 37.7% ± 7.6%; 24 h: 31.7% ± 5.7%; 48 h: 33.2% ± 5.7%) when compared to the DC-SIGN^Low^ population, independent of the time point evaluated (2 h: 11% ± 1.9%; p < 0.001; 24 h:12.5% ± 1.12%; p < 0.001; 48 h: 13.2% ± 3.6%; p < 0.001) (Fig. 4B). Nevertheless, the binding between Lb and DC-SIGN^Hi^ or DC-SIGN^Low^ did not present relevant differences (DC-SIGN^Low^, 2 h: 8.5% ± 1.16%; 24 h: 23.4% ± 4.28; 48 h: 35.3% ± 3.3%, and DC-SIGN^Hi^, 2 h: 12.8% ± 2%; p > 0.05; 24 h: 26.7% ± 5.5%; p > 0.05; 48 h: 37% ± 3%; p > 0.05) (Fig. 4C). However, in Lb, significant differences were observed when we analyzed the percentage of binding during the different times of interaction (DC-SIGN^Low^: 2 h vs 24 h, p < 0.05; 24 h vs 48 h, p < 0.05; 2 h vs 48 h, p < 0.001; DC-SIGN^Hi^: 2 h vs 24 h, p < 0.05; 24 h vs 48 h, p < 0.001; 2 h vs 48 h, p > 0.05) (Fig. 4C).

## DISCUSSION

In this study, we observed that American dermotropic species of *Leishmania* bind to DC-SIGN receptor with different patterns. La promastigotes bind to RAJI DC-SIGN^POS^ cells promptly, and the percentual of interaction increases according to the receptor expression levels. Lb promastigotes delay to bind to this receptor, binding with the same affinity to the DC-SIGN^Low^ and DC-SIGN^Hi^ population. Moreover, we found DC-SIGN positive cells in the inflammatory infiltrate of human CL lesions caused by *L. braziliensis*. Thus, it is possible that *Leishmania* uses DC-SIGN receptor to invade DCs or even to drive their functions.

Here we showed the presence of DC-SIGN^POS^ cells in CL lesions. It is the first description of presence of DC-SIGN^POS^ cells in CL lesions. Due to the cytoplasm characteristics, the histological aspects resemble macrophages. However, lymphocytes were also stained. In normal skin, DC-SIGN receptor is expressed in dermal DCs but not in Langerhans cells^25^ or in DCs expressing an immature phenotype.^14^ DC-SIGN receptor was discovered to bind DCs by the ICAM-3 and, for similar structure, to ICAM-2, but not to ICAM-1. The interaction DC-SIGN-ICAM mediates trans endothelial migration of DCs, from blood to tissues.^16^ DC-SIGN^POS^ cells presenting in CL lesions could be interacting with inflammed endothelial cells, especially those expressing ICAM-2 and ICAM-3, enabling them to exit skin and to home to secondary lymphoid organs. In other skin disorders, like leprosy and psoriasis, DC-SIGN^POS^ cells were already demonstrated in the inflammatory lesions.^19^ ^,^ ^20^ ^,^ ^21^ In leprosy, the presence of DC-SIGN^POS^ cells was associated to Th2 environment, being the major bacilli reservoir in lepromatous lesions.^19^ The function of DC-SIGN^POS^ cells in CL lesions is still unknown. It is possible that DC-SIGN^POS^ cells can be infected by *Leishmania* parasites. DCs with immature phenotype express DC-SIGN receptor and can harbor *Leishmania* parasites.^14^ ^,^ ^26^ Interestingly, when co-cultivated with polimorphonuclear cells, *L.* (*L*.) *amazonensis*-infected DCs exhibited lower rates of infection and parasite load, and this phenomenon seemed to be mediated by DC-SIGN^POS^ in a direct contact-dependent manner.^24^ This suggest that DC-SIGN^POS^ cells can uptake *Leishmania* amastigotes,^22^ corroborating with the hypothesis that DC-SIGN^POS^ cells in lesions can harbor *Leishmania* parasites. After infected, DC-SIGN^NEG^ cells monocytes-derivated reduce DC-SIGN expression,^24^ and are able to migrate to the lymph node, and then present antigens to naïve T cells.

Our results showed that promastigotes from both Lb and La species bind to the DC-SIGN receptor. After two hours of interaction, La is already bound to DC-SIGN receptor, while Lb takes longer to interact with DC-SIGN receptor, i.e., at least 24 h of co-culture. Those differences can influence the clinical and immunological outcome. Both Lb and La cause cutaneous form of leishmaniasis. *L. braziliensis* can cause a more severe and disfiguring form, mucosal leishmaniasis (ML). The cellular immune response to Lb infection is present and can be hyperactivated in ML.^27^ Indeed, there are rare and severe forms of La infection as diffuse cutaneous leishmaniasis, presenting nodular lesions whiting many infected macrophages, absence of cellular immune response and high antibodies titles.^28^ ^,^ ^29^ Dermotropic *Leishmania* species induce different patterns of DCs infiltration in lesions, as observed by others.^11^ In murine cutaneous lesions, La induces a rapid infiltration of DCs with a development of large lesions within many infected macrophages.^11^ *In vitro*, La is unable to activate DCs and induce cytokines production.^29^ Moreover, this parasite is capable of altering a DC differentiation associated marker.^30^ In the other hand, in Lb murine infection, DCs infiltrate coincides with lesion regression and increased number of infiltrating T cells.^11^ In DCs cultures with Lb, both DC non-infected and activated cells and DC *Leishmania*-infected and deactivated cells were found. Both DCs were influenced by *L. braziliensis* infection and contributed to control and immunopathology of the CL.^26^ Besides, it was shown that DC-SIGN mediates contact between human polimophonuclear cells and DCs, resulting in increased release of proinflammatory markers and reduced rates of La infection.^24^

Our results addressing American *Leishmania* species are in consonance with previous data, in which lower *L. major*-DC-SIGN interaction was observed in comparison to *L. pifanoi-*DC-SIGN.^23^ Here, we showed that the Lb binding to DC-SIGN receptor is delayed while La promptly binds to the same receptor. Moreover, La binds to the DC-SIGN^Hi^ population 3.5-fold more than to DC-SIGN^Low^. On the order hand, Lb promastigotes do not discriminate between the DC-SIGN^Low^ or DC-SIGN^Hi^ populations. Together, these results suggest that antigenic differences among the *Leishmania* species could be influencing the DC-SIGN receptor binding.

The present results extend the studies on the role of DC-SIGN receptor to species with epidemiological importance in the Americas. We showed, for the first time, that DC-SIGN positive cells are present in the inflammatory infiltrate of CL lesions caused by Lb. Curiously, although both Lb and La interact with DC-SIGN receptor, they differ in binding intensity and in the time for initiating the interaction. The exact function of DC-SIGN^POS^ cells in leishmaniasis has to be clarified, but our results indicate that *Leishmania* parasites can encounter this receptor in cells from infected skin and maybe they utilize it to infect DCs.
